# Cross-study analysis of genomic data defines the ciliate multigenic epiplasmin family: strategies for functional analysis in *Paramecium tetraurelia*

**DOI:** 10.1186/1471-2148-9-125

**Published:** 2009-06-03

**Authors:** Raghida Damaj, Sébastien Pomel, Geneviève Bricheux, Gérard Coffe, Bernard Viguès, Viviane Ravet, Philippe Bouchard

**Affiliations:** 1 Laboratoire Microorganismes: Génome et Environnement (ex. LBP) UMR CNRS 6023, Université Blaise Pascal, 63177, Aubière cedex, France

## Abstract

**Background:**

The sub-membranous skeleton of the ciliate *Paramecium*, the epiplasm, is composed of hundreds of epiplasmic scales centered on basal bodies, and presents a complex set of proteins, epiplasmins, which belong to a multigenic family. The repeated duplications observed in the *P. tetraurelia *genome present an interesting model of the organization and evolution of a multigenic family within a single cell.

**Results:**

To study this multigenic family, we used phylogenetic, structural, and analytical transcriptional approaches. The phylogenetic method defines 5 groups of epiplasmins in the multigenic family. A refined analysis by Hydrophobic Cluster Analysis (HCA) identifies structural characteristics of 51 epiplasmins, defining five separate groups, and three classes. Depending on the sequential arrangement of their structural domains, the epiplasmins are defined as symmetric, asymmetric or atypical. The EST data aid in this classification, in the identification of putative regulating sequences such as TATA or CAAT boxes. When specific RNAi experiments were conducted using sequences from either symmetric or asymmetric classes, phenotypes were drastic. Local effects show either disrupted or ill-shaped epiplasmic scales. In either case, this results in aborted cell division.

Using structural features, we show that 4 epiplasmins are also present in another ciliate, *Tetrahymena thermophila*. Their affiliation with the distinctive structural groups of *Paramecium *epiplasmins demonstrates an interspecific multigenic family.

**Conclusion:**

The epiplasmin multigenic family illustrates the history of genomic duplication in *Paramecium*. This study provides a framework which can guide functional analysis of epiplasmins, the major components of the membrane skeleton in ciliates. We show that this set of proteins handles an important developmental information in *Paramecium *since maintenance of epiplasm organization is crucial for cell morphogenesis.

## Background

Because of their asymmetrical and polarized cortical pattern, ciliated protozoans have long been interesting models for analysis of molecular mechanisms involved in cellular morphogenesis. *Paramecium *is a prominent example of an extremely organized membrane complex. The cortex is subdivided into hundreds of hexagonal cortical units, which give the cell surface a honeycomb aspect. Each unit is centered on a ciliary apparatus and encloses an alveolar sac between the cell membrane and the cytoskeletal material linked to the cytoplasmic face of the inner alveolar membrane[1-3]. Just beneath this inner membrane, lies a relatively thick and dense submembranous layer, the epiplasm, which surrounds basal bodies. The epiplasm can be either continuous along the anterio-posterior axis of the cell (*e.g. Tetrahymena*) or fragmented into adjacent scales, forming the cortical unit framework (*e.g. Paramecium*) [4]. To date, studies performed on the membrane skeleton of *Paramecium*, mainly *Paramecium tetraurelia*, were mostly descriptive, based on microscopic and biochemical analyses. The components of this sub-alveolar cytoskeletal material were shown to be numerous, with similar biochemical properties [4,5] and an antigenic heterogeneity at the level of epiplasmic scales [6]. Recently, it was demonstrated that the membrane skeleton of *Paramecium *is composed of similar proteins encoded by a unique multigenic family, the epiplasmins [7].

Given the rapidly increasing number of available organismic genomes, many potentially related sequences can be compared, and thus more integrative studies are emerging (for a recent review, see [8]). The *P. tetraurelia *genome has been duplicated three times and retains many duplicated sequences[9]. One of the major problems in analyzing a multigenic family is distinguishing duplications due to whole genome events from those resulting from segmental or single gene duplications. We here describe an updated analysis of the epiplasmin family of the ciliate *P. tetraurelia*, which regroups 51 proteins and attempt to correlate protein structure information obtained from Hydrophobic Cluster Analysis (HCA) [10] with both gene expression level and similarity tree analysis. Based on DNA sequence alignment and distance computation, a similarity tree groups the 51 epiplasmins in five main clusters. HCA reveals that the majority of the epiplasmin proteins obeys structural rules and can be represented as successions of modular structural domains. The analysis of the epiplasmin family of *Tetrahymena*, which comprises four members, is performed in parallel. A structural comparison of proteins from the five main groups in *Paramecium *with the related proteins of *Tetrahymena *demonstrates that they share the same global architecture, with a conserved central domain flanked by specific arrangements of common structural modules. In *P. tetraurelia*, according to the organization of the modules characterized by HCA, two symmetrical and asymmetrical classes can be defined among the groups of the similarity tree. Similarity and expression groups can further be correlated by regrouping epiplasmin genes according to the presence of TATA-like boxes and USE elements in the 5'UTRs. Preliminary RNAi experiments were performed to assess the biological significance of this classification.

## Results

### Updated phenogram of the epiplasmin multigenic family

**Figure 1:**
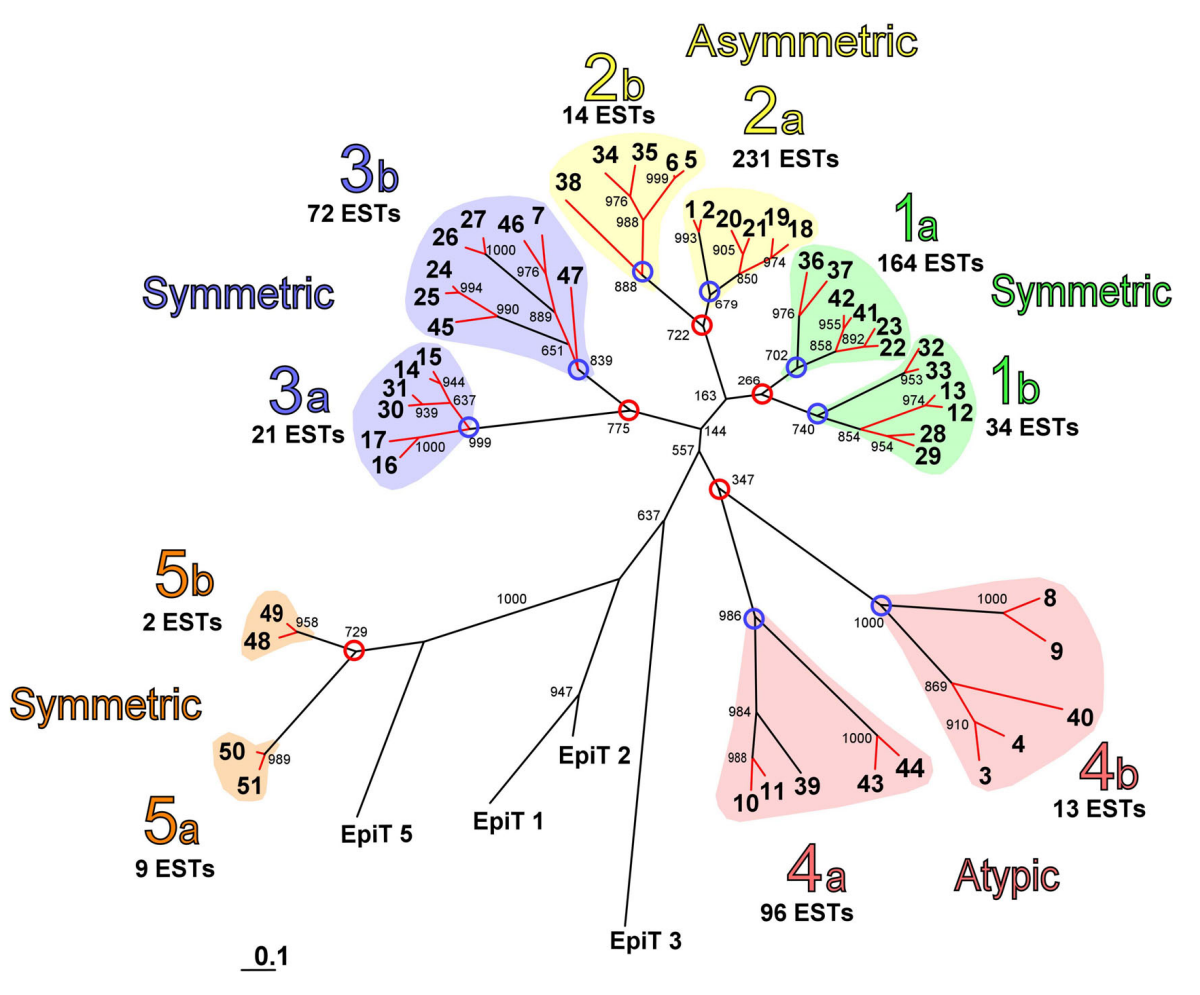
**Comparative analysis of *P. tetraurelia *and *T. thermophila *epiplasmins using DNA sequence alignment**. Five groups (1 to 5) were distinguished, based on node strength. According to hydrophobic cluster analysis, groups 1, 3 and 5 regroup 30 symmetric epiplasmins, group 2 represents 11 asymmetric epiplasmins, and group 4, 10 atypical epiplasmins. Results of large scale synteny analysis are reported as a red strand over the tree topology. Nodes corresponding to the 'ancient' and the 'old' WGD are circled in red and blue, respectively. EST numbers are compiled for each sub-group.

After completion of sequencing and annotation of the *P. tetraurelia *genome, 10 new related sequences (Epi 42 to Epi 51) of the epiplasmin family were found (Table 1), using a Blast search on the genomic bank (*Paramecium *DB [11]) according to that described in [7]. Similar Blast analyses were also performed on the recently released genome of another ciliate, *T. thermophila *(*Tetrahymena *Genome Database (TGD)[12] and two new sequences similar to *P. tetraurelia *epiplasmins were found, in addition to the two previously described [7]. These sequences are noted as: EpiT n (1, 2, 3 and 5). A CLUSTALW co-alignment was done with the 51 DNA sequences of *P. tetraurelia *and the 4 sequences of *T. thermophila*. Using the Phylip package, a similarity tree was created. As described in a previous work [7], since central domain of epiplasmins are conserved in EpiTs, EpiT 5 was used as an outgroup (Figure 1). This analysis, using the entire sequences, confirms the result previously obtained with the co-alignment of the central domains of the 41 previously identified epiplasmins, and completes the description of this family. In addition to the four main groups already described, a fifth group emerged including the four small sequences Epi 48, Epi 49, Epi 50 and Epi 51. Each of these five groups can be sub-divided in two sub-groups: a and b. Among groups 1, 2 and 3, each sub-group is composed of 6 closely related members which conform to a 4+2 topology, with the exception of sub-group 2b which has only 5 members, and sub-group 3b which has two additional orphan sequences. These groups, which compose 75% of the epiplasmin sequences, constitute a dense cluster clearly distant from groups 4 and 5. For *T. thermophila*, except for EpiT 5 which is closer to group 5, *T. thermophila *epiplasmins could not be clearly linked to groups 1 to 4. This shows strong sequence divergence between epiplasmins from the two ciliates, despite conservation of the central domains.

**Table 1:** Newly characterized epiplasmins (Epi and EpiT) and the three related proteins.

Bank ID	Protein	MW	pI	Group
GSPATP00038597001	Epi 42	30423	6.08	1a
GSPATP00017377001	Epi 43	24411	4.89	4a
GSPATP00019341001	Epi 44	41648	8.56	4a
GSPATP00007177001	Epi 45	26706	6.74	3b
GSPATP00010287001	Epi 46	26719	5.57	3b
GSPATP00017294001	Epi 47	26728	6.59	3b
GSPATP00018713001	Epi 48	16545	8.92	5b
GSPATP00027525001	Epi 49	17496	9.11	5b
GSPATP00032507001	Epi 50	20462	7.76	5a
GSPATP00033927001	Epi 51	20409	7.75	5a
6262	EpiT 1	32789	5.87	
6263	EpiT 2	32425	5.22	
14831	EpiT 3	23319	9.11	
20435	EpiT 5	19614	6.74	
2829	Tetra 2829	57966	9.34	
GSPATP00021655001	Para 21655	63754	6.68	
GSPATP00023342001	Para 23342	68117	6.76	

Within the tree, nearly all epiplasmins are represented by 2 copies issued from the recent whole genome duplication (WGD), except for 5 orphan sequences: Epi 38, Epi 39, Epi 40 Epi 45 and Epi 47. In good agreement with the tree topology, the gene relationships (Figure 1, red links) inferred from a large scale synteny analysis[9], define paralogous sequences linked through several successive nodes corresponding to at least three genome duplications. Nodes at the root of sub-groups 3a and 2b (Figure 1, blue circles) correspond to the 'old' WGD event defined in[9]. The other sub-groups presenting the same topology could be also associated to the 'old' WGD event even if this is not supported by synteny analysis. Nodes separating these sub-groups (Figure 1, red circles) could relate to a more ancient event not necessarily linked to a large scale genome duplication.

### Hydrophobic cluster analysis (HCA) of the epiplasmins

The previous analysis, based on sequence similarities, grouped 75% of the epiplasmin sequences in a dense crown composed of 6 sub-groups. This computed topology does not yield relevant information on features specific to each of these groups. We used HCA to determine the structural characteristics of each epiplasmin.

HCA is a powerful method to produce information using graphical and bi-dimensional representation of proteins [13]. The technique is useful to delineate protein domains, and to predict structural properties. This approach permits comparison of proteins which have become widely divergent (less than 30% identity), and/or proteins with large domain insertions or deletions. In HCA protein comparison, one initially considers general conservation of hydrophobic cluster shape, which can be altered by substitution of non-hydrophobic residues in place of hydrophobic residues. In this case, the behavior of these non-hydrophobic residues is dependent on the context of their 3-dimensional environment. In a mainly hydrophobic region, residues such as A, S, T, C and even Q behave as hydrophobic, and thus act as mimetic residues.

The recurrent presence of several clusters defines and affirms the existence of a structural domain with conserved features, even when corresponding sequences show less than 30% identity. Similarly, the determination of conserved domains is not affected by insertions or deletions of any size. HCA comparison also permits the identification of invariant residue positions. Such topological positions reinforce the overall similarity of clusters and thus of structural domains.

**Figure 2:**
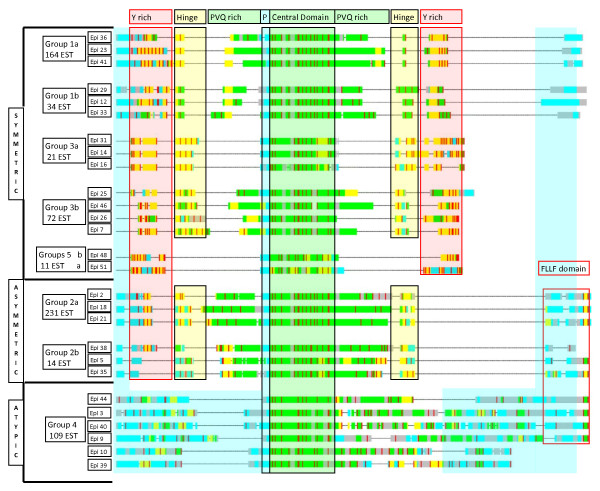
**Modular organization of the *P. tetraurelia *epiplasmins, using generalized cluster analysis representation**. For each protein, hydrophobic, loop and ambivalent clusters are represented by green, blue, and yellow blocks, respectively. Several structural domains, determined by HCA, are boxed in green for the central domain shared by the whole set, in yellow and pink for the hinge and Y rich domains, respectively.

The complete HCA representations of the 51 epiplasmin proteins are presented as supplementary data [see additional file 1]. Analyses of these schematic representations demonstrate specific domains within each epiplasmin. For clarity, these data are presented according to Generalized Cluster Analysis (GCA) in Figure 2.

The HCA technique was initially based on the assumption that clusters of hydrophobic residues can constitute the internal core domain of a protein, thus minimizing interaction with solvent. HCA was later extended to GCA, using the same approach but including clustering of 'loop forming residues' with the propensity to interact with solvent. The combination of the two cluster types allows classification of proteins as modular assemblies of structural domains. Figure 2 presents a sampling of the 51 epiplasmins that considers only one representative of the last WGD, except for group 3b. In this case, genome duplication history for Epi 46, 47, and 7 is not in agreement with their structural features. According to synteny analysis, Epi 46 and 7 are paralogs from the recent WGD. They present however significant structural differences while Epi 46 and 47 are structurally strongly related. In order to illustrate the extended modularity of the epiplasmins, Epi 7 was chosen in place of Epi 47.

Characteristic features, as determined by HCA, are represented using GCA and the presentation highlights different structural domains in which hydrophobic, loop and ambivalent clusters are represented in green, blue, and yellow, respectively. The main feature characterizing these proteins is a strongly structured central domain, which begins with a short unstructured motif containing a serine/threonine phosphorylation site. On each side of the central domain, the N- and C-term arms contain several structural domains: i) a structured domain containing repetitive PVQ rich motifs, ii) a hinge with a Ph [QSTR]Y [AS] consensus indicative of beta turns or, simply, of a transition between two domains, iii) a Y rich domain. Epiplasmins can thus be grouped by module similarities and arrangement. This structural grouping matches the tree topology obtained by comparison of aligned sequences, including the partition of sub-groups a and b.

Most epiplasmins (41) present a sequential arrangement of these structural domains, and can be classed as symmetric or asymmetric. In contrast, the 10 remaining epiplasmins (group 4) are characterized by an abrupt transition on each side of their central domain, with two rather unstructured (random coil) N-left and C-right terminal arms. These proteins constitute a separate atypical class.

Among the symmetric epiplasmins, structural domains are disposed as a mirror image on each side of the central domain. The symmetric proteins of groups 1 and 3 have a complete set of domains: one PVQ rich, a hinge, ending in a Y rich domain. Proteins from these groups can be distinguished by major structural differences. In group 1, the PVQ rich and Y rich domains are separated by only one hinge sequence. However, in group 3, the hinge is duplicated 5 to 6-fold, and sometimes overlaps or tends to mimic a PVQ rich domain (absent in sub-group 3a). In addition, proteins from sub-groups 1a, 1b, 3a, 3b are distinguishable. Epiplasmins from sub-groups 1a and 1b differ in their Y-rich regions. In sub-group 1a, proteins present clear harmonics (repetitive occurrences) of tyrosine residues while proteins of sub-group 1b present a more disorganized pattern of tyrosine residues. Epiplasmins of sub-group 3a differ from those of sub-group 3b by the absence of PVQ-rich regions. Group 5 proteins are symmetric but less complex, with only the Y rich sequences on both sides of the central domain.

In the asymmetric class, represented by the group 2, sequential domain arrangement is conserved in the N-term arm, whereas the C-term arm presents an alternative domain. The hinge domain is followed by a rather unstructured sequence ending in a very specific motif of hydrophobic and aromatic residues, which constitutes a conserved C-term FLLF cluster, defining a new domain that we call 'FLLF' domain. Proteins of group 2a differ from those of 2b by harmonic distribution of tyrosine residues in their Y-rich regions.

The FLLF domain is not limited to asymmetric epiplasmins; it is found in some proteins of group 4 (Epi 3, 4, 8, 9, 40, 43, 44) which lack readable geometry.

The HCA approach, by enabling the comparison of sequences with large insertions or deletions, demonstrates that epiplasmins are modular proteins.

### Screening for proteins containing epiplasmic-related domains

**Figure 3:**
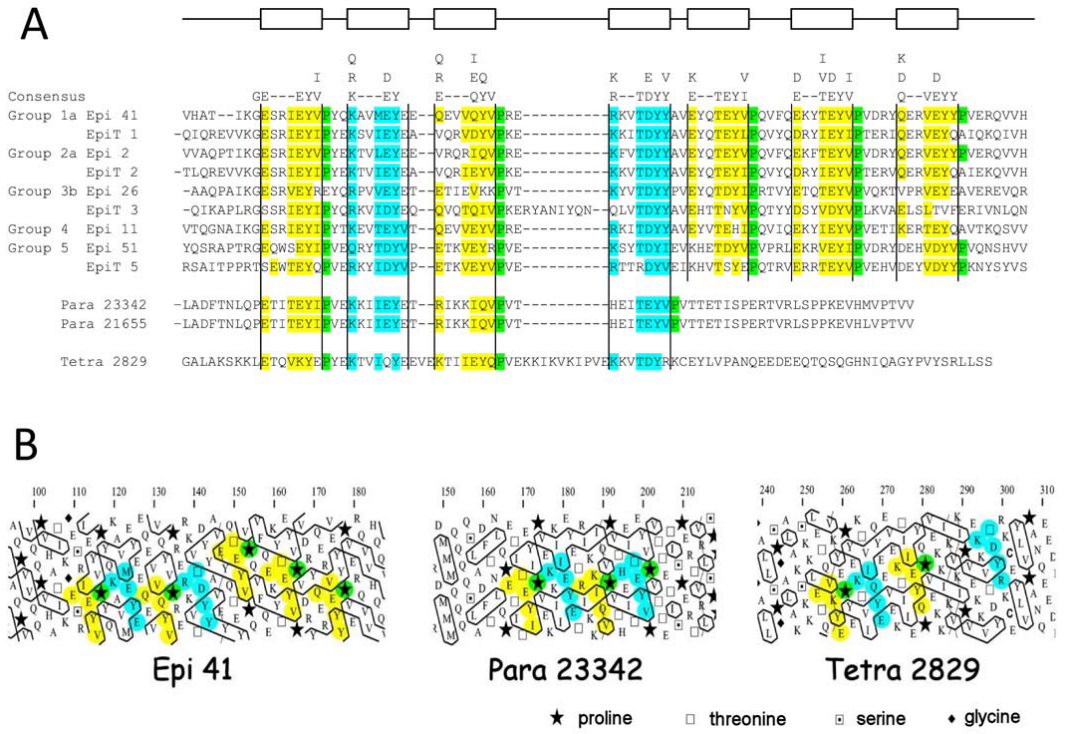
**Modular organization of the central domain of epiplasmins**. A-Alignment of the seven repeated motifs in central domains of *P. tetraurelia *and *T. thermophila *epiplasmins. Two paralogs in *P. tetraurelia *and one *T. thermophila *protein share the first half of the epiplasmin central domain. B-HCA representation of these motifs is used to consider the position of conserved amino acids in relation with the hydrophobic clusters. This shows a spatial proximity between conserved proline and tyrosine (as mentioned in the discussion).

Refined analysis of the central domain common to all epiplasmins demonstrated that it is composed of seven repeats of the following consensus motif: [ERK]xx[VILT]EY[VIY] (Figure 3A). This motif can also be observed in the central domain of *T. thermophila *EpiT 1, 2, 3 and 5 (Figure 3A).

A BLAST search for this motif in the *P. tetraurelia *genome, associated with an HCA analysis of each relevant result, yielded a pair of paralogous proteins "Para 21655" and "Para 23342". The core domains of these proteins contain the first 4 of the 7 motifs. Screening of the *T. thermophila *genome revealed a protein we called "Tetra 2829", with the same 4-motif pattern (Figure 3A and 3B, and Table 1). Although these *T. thermophila *proteins are quite divergent from the *P. tetraurelia *epiplasmin family in their N and C-term sequences, they present the same overall abundance in P and Q residues.

The discovery in *P. tetraurelia *of two genes encoding for proteins that share half of the epiplasmin central domain, and one in *T. thermophila*, reinforces the utility of cytoskeletal proteins as evolutionary markers, and thus requires a closer examination of the relationship between EpiT proteins and the *P. tetraurelia *epiplasmin set.

### Comparison of *Paramecium *epiplasmins with their related proteins in *Tetrahymena*

**Figure 4:**
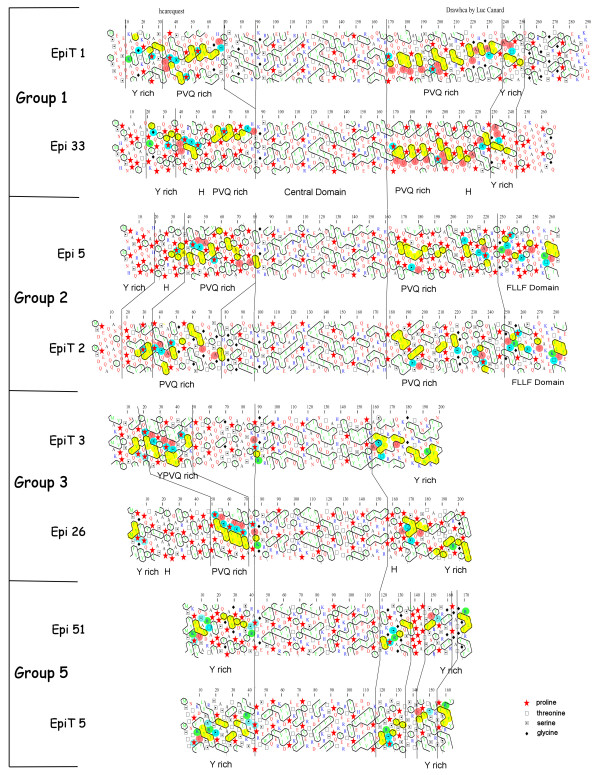
**Evidence for common structural domains between *P. tetraurelia *epiplasmins (Epi) and *T. thermophila *epiplasmins (EpiT)**. HCA representation, where hydrophobic residues are shaded in yellow; helix breakers P, G, S, T in blue; alkaline residues in green and A, N, Q, D, E in pink.

The HCA approach was used to determine whether these proteins also show a modular organization. In Figure 4, we show structural comparison of EpiT 5, the smallest *Tetrahymena *protein, with Epi 51, one of the smallest group 5 epiplasmins. Both display an initial hydrophobic cluster surrounded by specific conserved basic residues (R/K). Downstream, successive clusters exhibit numerous Y residues and finish by a conserved group of S, R, T, P residues. The overall distribution of hydrophobic clusters and conserved residue positions show that these two proteins share a similar structure. For the C-term part of the protein, a group of R, S, Y residues after the position 120 of each protein was used as an anchoring (meaning "starting") point. This cluster is followed by a set of proline residues in Epi51 and a set of serine residues in EpiT 5. Both serine and proline are structural breakers. A final Y rich cluster is evident, with two topologically conserved arginines. It thus appears that both epiplasmins are structurally similar and share symmetry, with a central domain flanked by two Y rich domains.

In EpiT 3, compact hydrophobic clusters with a high content of aromatic residues are observed in the right arm of the protein, as in the group 3 of epiplasmins in *Paramecium*. The residues at positions 160–174 in EpiT 3 are identical to those of Epi 26. (These residues in a conserved structure are termed topologic residues). Even if hinge domain is not recognizable in EpiT 3, we can demonstrate that both proteins end with a Y rich domain or more generally by an aromatic rich domain (in EpiT 3 Y residues are often replaced by F residues). Epi 26 begins its N-term with a peculiar hydrophobic cluster followed by 2 prolines. This constitutes an anchor for analysis which can be found at position 13 of EpiT 3.

In both proteins a group of topologic residues (P, A, [I, L], [R, K]) finishes the N-term domain. Between these two reference points, the sequences share a PVQ rich stretch. In EpiT 3, this cluster is between positions 20–40. In Epi 26, from 50–71. In EpiT 3 this PVQ rich domain is connected to the central region of the protein by a less structured stretch, while in Epi 26 this stretch is upstream of the PVQ rich domain.

Despite the group 3 heterogeneity, this analysis of EpiT 3 indicates that this protein is structurally close to group 3 epiplasmins.

The affiliation of EpiT 2 and group 2 epiplasmins is more evident. The typical alternative domain observed in asymmetric epiplasmins is present in EpiT 2 with the terminal FLLF cluster. We chose Epi 5 as a representative of group 2 epiplasmins in *Paramecium*. At the N-term, these proteins begin with an unstructured 20 amino-acids long sequence followed by a hinge domain. A similar P, V, Q rich region is evident, beginning with a group of topological residues (P, Q, S, A) at position 40 and visible until the 80th position.

EpiT 1 harbors two PVQ rich domains flanking the conserved central domain. A repetitive occurrence of VQQ or PVQ sequences is noticeable with an organization of hydrophobic clusters. No FLLF domain is present, as previously observed for symmetric epiplasmins assigned to group 1. Epi 33 matches the distribution and shape of EpiT1 hydrophobic clusters. Two terminal Y rich domains are seen at both ends of the proteins.

This comparison of *Paramecium *and *Tetrahymena *epiplasmins shows the relation between groups 1, 2, 3, and 5 to EpiT 1, 2, 3, and 5, respectively. HCA demonstrates the clear similarity and geometric conservation of proteins sharing less than 15% identity beyond the common central domain.

### Expression and putative promoter elements

Taking advantage of the automatic annotation of the *Paramecium *genome, a compilation of the number of ESTs for each of the 51 epiplasmins was performed (from file ), giving a probable estimate of their expression rate (within a range of 0 to 72 EST).

In the partitioning of these 656 ESTs within the similarity tree topology, a very uneven distribution between groups and sub-groups is visible: over 71% of ESTs concern only 3 sub-groups: 1a, 2a, 3b (Figure 1). To determine if this unequal partitioning of ESTs between sub-groups a and b indicates a differential regulation of expression in *Paramecium*, we thus examined the 5'UTR of the corresponding genes.

The promoter region of ciliate genes remains poorly characterized. We sought specific promoter elements in upstream regions of DNA sequences. The epiplasmin multigenic family gave us the opportunity to seek, in several related genes, consensus motifs in the upstream regions.

The short macronuclear intergenic regions in *Paramecium *facilitate the search for putative regulating sequences. The 5'-UTR of epiplasmin genes classified by the number of reported ESTs, followed by respective group memberships, were then manually aligned to tentatively find conserved motifs.

**Figure 5:**
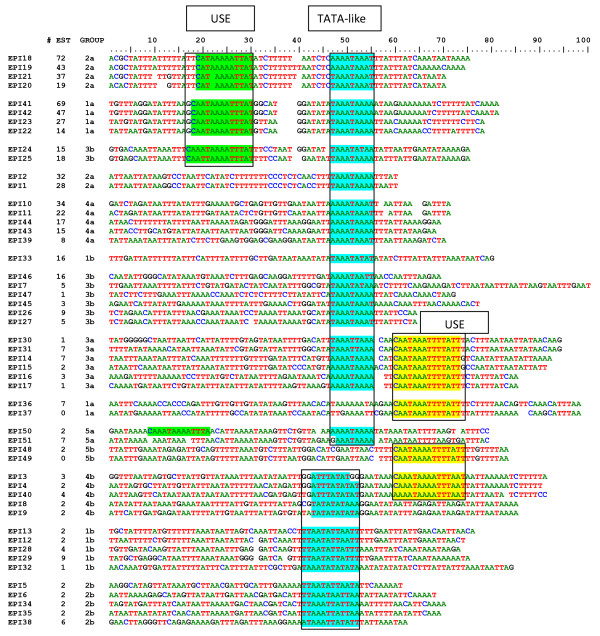
**5'UTR analysis of *P. tetraurelia *epiplasmin genes**. The genes are regrouped according to EST numbers and presence of USE and TATA-like elements. These motifs are shaded in blue for TATA-like element, and in green or yellow for the USE motif according to whether it is located upstream or downstream from the TATA-like element, respectively.

As shown in Figure 5, most of the epiplasmin genes present a common AT-rich stretch, 5 to 45 bp upstream of the ATG with the main motif [AT]AAATAAA [AT] resembling a TATA-box element. When this motif is slightly degenerated, it appears that nearly all genes (except Epi 36, 37, 48, and 49) present a putative TATA-box element. An additional motif, 12 to 20 bp upstream of the TATA-like motif with a CA(1,3)TA(3,4) [AT]TTAT consensus is seen. This upstream sequence element (USE), in the 10 first lines (green background), is present in sequences from sub-groups 1a, 2a and 3b. These 10 sequences comprise 55% of ESTs seen in the entire set of epiplasmins.

This USE element (yellow background) can be found 3 to 5 residues downstream of the TATA-like box. As no corresponding sequence is associated with a high number of ESTs, a 'misplacement' of the USE motif may abolish its putative enhancer role. All members of sub-group 3a share this misplacement and could act as a weak expression group. By contrast, genes of sub-group 3b do not present a homogeneous regrouping of their 5'UTRs.

### Epiplasm alteration by specific RNAi: Local and global effects

Additional results of the analysis of the multigenic family of epiplasmins using similarity tree, structural and transcriptomic approaches clearly demonstrate the importance of sub-groups 1a and 2a. They are structurally homogeneous, representative of symmetric and asymmetric classes and account for 60% of reported ESTs. We thus selected representatives of the family from these groups for functional investigations.

We used the Epi 2 and Epi 41 sequences, of asymmetric and symmetric classes, for which 32 and 69 ESTs were found, respectively. RNAi was obtained by feeding *Paramecium *with *E. coli *HT115 expressing the whole coding sequence of Epi2 or Epi41 as dsRNA.

**Figure 6:**
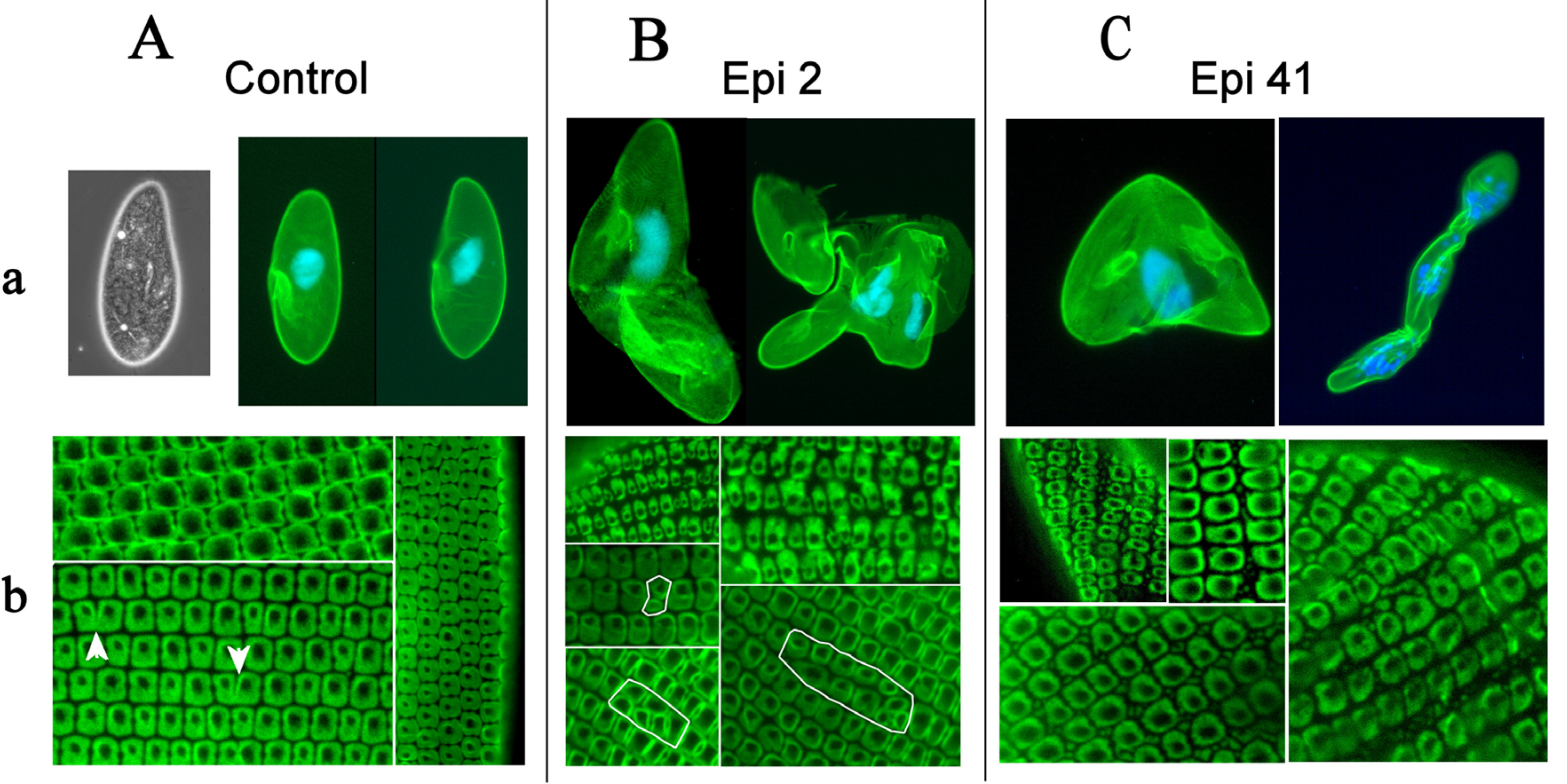
**Effects of RNA interference of genes coding for asymmetric and symmetric classes of epiplasmins on *P. tetraurelia***. A-Control Cells: a) Low magnification phase contrast and immunofluorescence with mAb CTS32 counterstained with DAPI. b) Higher magnification showing the alignment of cortical units along the kinetids. The arrow heads show normal pattern of scale duplication. B-Cells submitted to Epi 2 RNAi: a) Cell shape alteration at division time (boomerang) and 24 h later (plasmodial form). b) Irregularities in the alignment and the shape of the scales, and abnormally oriented striction of scales leading to 'double kinetids' (circled with white line) C-Cells submitted to Epi 41 RNAi: a) Same cell shape alteration as with Epi 2 at division time (boomerang) and 24 h later (plasmodial form). b) Numerous granules of epiplasmic material surround crenulated scales.

The RNAi time course was followed *in vivo *by light microscopy and by fluorescence microscopy after immunostaining, using the CTS32 antibody. This mAb recognizes the whole set of epiplasmin proteins [4] and can be used as an epiplasm marker. During the first hours, RNAi of either Epi 2 or Epi 41 triggers an alteration of cell shape, the loss of their slenderness: cells round up except at the anterior pole, giving rise to pear shaped cells, a phenomenon also described for some actin RNAi experiments [14]. At the time of division, cytokinesis is impaired and cells appear as "boomerang" shaped (Figure 6 Ba and Ca). Plasmodial forms accumulate 24 h to 48 h after feeding, due to repetitive aborted divisions. Otherwise, RNAi of Epi 2 or Epi 41 does not affect cell viability. Ciliary movement does not seem to be altered, as we have observed that cells are able to feed on "Chinese" ink (data not shown). Swimming patterns seem normal, unless cell geometry is modified. Contractile vacuoles and gullets duplicate and appear functional.

Immunofluorescence analysis using the CTS32 antibody was used to see if this phenotype resulted in marked reshuffling of epiplasmic material. Despite the severity of the phenotype, observation of fixed cells immunostained by CTS32 show no difference in the intensity of the fluorescent signal, or major relocalization of epiplasmic material within the cells. Unlike the unique global phenotype, each Epi 2 and Epi 41 RNAi elicits specific local alterations of this submembranous cytoskeletal component.

Epi 2 RNAi induces an alteration in the duplication of epiplasmic scales resulting in either unequally sized cortical units or aberrantly oriented (antero-posterior) segmentation of the epiplasmic scales, leading to local doublings along the kinetids (Figure 6Bb). In cells submitted to Epi 41 RNAi, CTS32 mAb decorates numerous nodules of epiplasmic material which appear between crenulated cortical units (Figure 6Cb).

## Discussion

### Agreement phylogeny/HCA

While not commonly used in phylogenetic studies, HCA is useful in defining structural similarities between sequences with low identity levels (<30%) and/or variations in inter-motif distance [15-17]. After the *Paramecium *genome was sequenced, the multigenic epiplasmin family was reevaluated as containing 51 members. As seen in figure 1, sequence information alone is not sufficient to infer with success relationships between *Tetrahymena *and *Paramecium *representatives. Since protein structure is much more evolutionarily conserved than sequence, the similarity tree based on co-alignment of the entire sequences was compared to structural analysis of protein domains obtained using the HCA method. A recent work by Silva [18] uses an algorithm (based on an HCA representation of proteins) intended to automatize a large scale databank screening, defining TM-scores to select with a good probability a group of proteins sharing structural homology. Proteins analyzed by Silva are mainly composed of globular domains. This analysis does not consider proteins with extended helices such as coiled coil or proline helices. Such features can be found in epiplasmins. In the present study, the use of HCA was to decipher domains for comparison of proteins, in relation to the similarity tree.

The combined results confirm the classification of these 51 epiplasmins in 5 groups. All the proteins share the highly conserved central domain previously described [7], which constitutes the signature of this family. While an apparent variability can be seen in the flanking N- and C-term sequences, it is possible through HCA to characterize a set of various structural domains (PVQ-rich, Y-rich, Hinge, FLLF-motif) which constitute the proteins. The different arrangements of these building blocks leads to a classification in quite good agreement with the clustering obtained with the similarity tree. The family can be divided in 3 classes: symmetric, asymmetric and atypical. The symmetric class comprises 30 proteins with structural domains disposed in mirror image on each side of the central domain. This class is composed of three groups, groups 1, 3 and the more distantly related group 5. The four epiplasmins from group 5 are the smallest of the family, having kept only a Y-rich domain on each side of their central domain. The asymmetric class, group 2, has 11 members. These proteins retain the same structural domain organization as the symmetrical proteins in their N-term arm but their C-term region has lost the Y-rich module. The atypical class, group 4, contains 10 proteins. It differs from the two other classes in an absence of common modules in the N- and C-term regions but possesses a FLLF domain in common with the asymmetric group. This FLLF domain could likely be involved in specific interactions within the epiplasmic structure.

### Expression groups and genome evolution

According to the epiplasmin-specific ESTs retrieved from *Paramecium *DB, nearly all members of the multigenic family are expressed. Within this multigenic family, maintenance of genes during the successive genome duplications is in good agreement with expected behavior of genes subject to dosage constraints (highly expressed genes are preferentially retained as duplicates[9]). Most of the sub-groups observed in the epiplasmin family have a 4+2 topology, a distribution also described for other sets of highly expressed proteins: 35 cGMP-dependent protein kinases [19], 26 syntaxins [20], 12 synaptobrevins [21] and 17 vacuolar-proton-ATPases [22].

Regarding the distribution of the 656 ESTs retrieved from *Paramecium *DB, one can see that gene expression levels are not evenly scattered over the similarity tree topology. It appears that nodes (Figure 1, red circles) separating the five groups into sub-groups a and b insure not only a partition between expression groups, but also between groups of genes which possess or lack recognizable motifs within their 5'UTRs. These nodes could be associated with an event of regulatory motif modification in the 5'UTR of sub-group b genes. After duplication, *Paramecium *may have maintained one of the genes and modified the 5'UTR of another, providing the opportunity to neo-functionalize.

For example, proteins of sub-group 2a are structurally identical; their genes possess both USE and TATA-like motifs, and are highly expressed. Members of sub-group 2b also share evident structural identity, thus suggesting a very similar function within the epiplasm, while none of the genes 2b presents USE/TATA-like motifs in their 5'UTR. The large number of EST found in group 2a suggests that the epiplasmic protein function has an important (stoichiometric) need for asymmetric forms of epiplasmin proteins. In contrast, group 2b may have been maintained because they have diverged to perform a new function that does not require that high levels of the protein be present.

### Expression groups and putative promoter motifs

Other multigenic ubiquitous genes like actin or tubulin have apparently undergone an extensive process of neo-functionalization[14,23]. For the epiplasmins, this process is not very evident, but the co-existence of sub-groups possessing or lacking putative regulation elements could constitute evidence of sub- or neo-functionalization. For instance, highly expressed sub-groups 1a and 2a could be used in a constitutive way all along the cell cycle, whereas sub-groups 1b and 2b would be expressed only at a precise point in the cycle.

Three distinct types of region are thought to constitute eukaryotic promoters: TATA boxes, CCAAT boxes and GC boxes [24]. Although the sequence TATA [TA]A [TA] is the consensus eukaryotic TATA element in most eukaryotes, it has been demonstrated that most AT-rich sequences of 6 base pairs or longer can convey TATA activity in the proximity of other control elements. The promoter region of ciliate genes is presently poorly characterized [25]. A study of a set of homologous intergenic regions from 22 species of *Tetrahymena *reveals a single putative promoter element, with a consensus sequence TATCCAATTCARA. This sequence contains a 'CCAAT' box, which also occurs at 8 locations preceding other ciliate genes and no 'TATA' box was found [26]. Recently, the MTT2 gene of *Tetrahymena thermophila *was shown to contain regulatory elements such as a TATA-box, CAAT-box and several MRE-like and ARE-like sequences [27]. In the ciliate *Stylonychia lemnae*, the study of the α-tubulin minichromosome revealed a TATA-like element, a putative initiator element and two distinct upstream sequence elements (USEs) [28]. The short 5' non-transcribed spacers between *Paramecium *genes offer an extraordinary opportunity to study transcription initiation in these organisms. To date, in *Paramecium*, sequence comparisons have not identified highly conserved promoter sequences but the presence of TATA or CAAT boxes is suggested. We here provide an analysis of the epiplasmin family on the presence of putative TATA-like elements and upstream sequence elements (USE) in relation with the expression levels of the corresponding gene products.

This analysis leads us to propose that an association of the USE and TATA-like motifs could constitute a strong promoter region, since the ten sequences presenting this association represent only 1/5 of the 51 epiplasmins, but are responsible for as much as 55% of the EST observed for this family. The functional significance of these elements is unknown, and this opens an interesting field in Ciliates gene regulation.

### Functional analysis by RNAi

RNAi experiments were conducted with representatives from sub-groups 1a and 2a. Sequence homology, at the 23mer level, [see additional file 2] within these sub-groups cannot preclude that RNAi conducted with of one sequence also blocks the expression of the other genes in the same sub-group: Epi 2 RNAi could block the expression of Epi 19 since this sequence has 10 common 23mers with Epi 2; similarly, Epi 41 RNAi which could block the expression of Epi 23 within the group 1a (23 common 23mers). In contrast to this non-selectivity of RNAi within a sub-group, Epi 2 and 41 do not share any common 23mer; they were chosen for their inter-group selectivity. The observed distinct local effects induced by the Epi 2 and Epi 41 RNAis show the group specificity of the triggered interference mechanism. These experiments suggest that both symmetric and asymmetric classes of epiplasmins are required for proper epiplasmic scale formation.

Moreover, blocking expression of these epiplasmins, although group specific, yields a common global cellular response; a change in cell shape, followed by impaired cell division and the appearance of 'monstrous' plasmodial forms, without loss of viability as some experiments were conducted for 2 weeks. This monstrous "pluricellular paramecium" resembled a plasmode. As development goes, there is proliferation of individual organelles (contractile vacuoles, gullets, nuclei) within one global plasma envelope, cohesiveness of these forms becomes too weak in relation to the size of the plasmode. Then plasmode explode under observation between slide and cover slip. We were able to count at least 32 gullets, while 64 were suspected but not demonstrated.

Abnormal or 'monstrous' phenotypes were described as early as the turn of the past century, following merotomy experiments [29], or chemical exposure to agents such as an insecticide, benzene hydrochloride [30], or by x-rays [31]. A similar phenotype, characterized by impaired cytokinesis and abnormal macronuclear division, was reported after treatment of *Paramecium *by 6-DMAP, an inhibitor of serine-threonine kinase activity [32]. These phenotypes are probably due to the numerous phosphorylable molecules targeted by this drug. In regard to the conservation of a potential serine-threonine kinase site in the central domain of all epiplasmins [7], we could propose that these proteins are part of the many molecules affected by the drug.

More recently, the use of RNAi, a supposedly more specific tool acting on one target protein, permits a less complex interpretation of phenotypes. Cytokinesis impairment has been described in an exhaustive study of the actin paralogs in *Paramecium*.

As cited in [33], cells expressing a construct GFP-act4 "did not divide after microinjection and were too sensitive for observation under the microscope". Moreover, the invalidation of the actin4 gene can lead to an altered cell division with the formation of a boomerang shaped cell. This effect is sequence-specific as none of the RNAi with the other 24 actin genes can trigger this phenotype. It is noteworthy that in the boomerang cells induced by actin4 RNAi, the exocytotic capacity is reduced to 10%, showing the role of this actin4 in the docking process of the trichocysts to the membrane. It would be of interest to check whether epiplasmin RNAi affects or not exocytosis since both actin and epiplasmin experiments trigger an alteration localized at the cell surface. Considering the lack of trichocyst anchoring observed from actin RNAi, the flattening of the epiplasmic scales observed from epiplasmin RNAi, and the weakening of the cell resistance to deformation by pressure observed from both actin and epiplasmin RNAi, it is likely that these various effects reflect an alteration of one cortical attribute: its tensegrity. We propose that the integrity of the cortical tensegrity system is required to keep and transmit a structural information content 'coding' for a positional blueprint of cell division. As epiplasmins form a major part of the cortex and are involved in its integrity, they probably play a role in the transmission of this type of 'epigenetic' information. These proteins, previously identified by cell fractionation, show in vitro reassembly in large fibrillar complexes [5]. Regarding the set of epiplasmin sequences, one could search for potential protein-protein binding sites which account for this copolymerization capacity. In the different domain arrangements within proteins from groups 1, 2, 3, and 5, searching for a minimal essential composition required for epiplasmic function, one can see that PVQ rich and hinge domains could be dispensable (group 3a, group 5) while Y rich domains are present in all 41 proteins. The sequences corresponding to these domains present an alternation between tyrosine and proline residues, with 2 prevalent motifs: P-X-Y and P-h-X-Y (with h for hydrophobic residue). Even the 10 sequences from group 4 present these motifs although no Y rich domain can be identified. These proline rich regions (PRRs), albeit not strictly organized in tandem repeats, have been described as involved in protein-protein binding [34,35]. The PRRs (proline rich regions) have been described as interacting with aromatic residues, as is the case for a PRR from the 3BP-1 protein with the SH3 domain of the Src protein kinase, which has a binding site lined with conserved tyrosine and tryptophan residues [35]. The PRR proline-rich salivary protein has also been shown to interact with polyphenols (tannins) via proline residues [35]. The Y rich domains present in most of the epiplasmins could provide them with a protein-protein binding capacity based on reciprocal inter-chain proline/tyrosine C-H...π interactions[36]. Due to the permanent constraint on the peptide backbone conformation of prolines, these stretches could present more rigid segments having a lower entropy loss upon binding. Repetition of several alternated tyrosine and proline could be a way for increasing weak individual bindings, in much the same way as additive H-bounds can stabilize double-strand DNA.

RNAi experiments in *Paramecium *demonstrate the crucial importance of a functional epiplasm for the maintenance of a normal development. HCA analysis of the two sets of epiplasmins in *Tetrahymena *and *Paramecium *indicates that both species harbor 4 to 5 main representative classes composed of proteins having specific structural architectures. Such sets of proteins have not yet been discovered in other ciliates. It is therefore not possible to determine if these two ciliates share an epiplasmic set of protein issued from a common ancestor or from functional convergence. EpiT localization and function in *Tetrahymena *will bring more information in order to evaluate the significance of these proteins in cell morphogenesis.

## Conclusion

In this report, the genome biology of two ciliates was studied using several independent methods. *Paramecium *genome duplications offer the opportunity to examine the divergence and functionalization of genes within a single cell. The cell provides a simple evolutionary laboratory and allows an internal control of tools used to compare distant sequences. The simultaneous usage of phylogenetic algorithms and HCA aids in defining the organization of a multigenic family, here the epiplasmin family. We now have a working framework for functional analysis. It addresses the presence of putative regulating sequences for gene expression. From RNAi experiments it demonstrates that epiplasmins are of critical importance for cell morphogenesis. Last, it postulates that structural diversity between epiplasmins would reflect various functions.

Our results enlarge the epiplasmin multigenic family organization to another ciliate genus, *Tetrahymena*. The distinctive identification and classification of epiplasmins open the way towards a better-defined functional analysis in *Paramecium *and *Tetrahymena*. It also opens questions about cortical morphogenesis in these two ciliates.

## Methods

### Cell cultures

The wild-type strain, *Paramecium tetraurelia *8-2B, was grown at 28°C in grass infusion medium WGP (Wheat Grass Powder, Pines International, Lawrence, KA) infected with *Klebsiella pneumoniae *the day before use and supplemented with 0,4 μg/ml β-sitosterol, as previously described [37].

### DNA and amino-acid sequence analysis

Epiplasmin nucleic sequences [see additional file 3] were aligned by the CLUSTALW (v1.8) program available at , and processed through the Phylip 3.6 package  to build a similarity tree; distances were calculated by DNADIST using the F84 model. Trees were elaborated by the FITCH program using the Fitch-Margoliash method using the G (global) search parameter. Confidence levels were evaluated using 1000 bootstrap replicates and 10 jumbles for each replicate. The rooted tree was drawn with Treeview v1.66 software, available at . Hydrophobic cluster analysis (HCA) of epiplasmins was performed according to [10]. Graphical representations were obtained from the RPBS service at . Generalized cluster analysis (GCA) was used to discriminate the different structural domains according to their propensity to form loops or alpha-helices, using a three-residue threshold for cluster validation, a connectivity distance of four, and the hydrophobic and loop alphabets described in [13].

### DNA cloning and sequencing

Epi 2 and Epi 41 sequences were amplified by PCR on genomic DNA using specific primers: Epi2S: 5'-AATAAATCTAGAATGAGCAATATCCCACAATCA-3' and Epi2AS: 5'-TTATGAGGGCCCATCTCTATCAAAGAGTCTGTC-3' for Epi 2, and Ep41N: 5' GCGGCGAATTCATGTCACAAAGACCACCCGTC 3' and Ep41R: 5' GCGGCGAATTCTCTGGGAGGTTATTATTAAAC 3' for Epi 41. Amplification products were cloned in the Litmus 28i plasmid (New England Biolabs) and transfected in competent HT115 bacteria designed for feeding-mediated RNAi [38]. Plasmid inserts were sequenced on both strands using Litmus specific primers and the DTCS Quick Start Kit (Beckman Coulter, France).

### Feeding-mediated RNAi

Feeding experiments were performed as described [38]. Negative controls fed *P. tetraurelia *with HT115 bacteria transformed only with the Litmus plasmid.

### Microscopy

Cells were permeabilized for 1 min using 1% Triton X-100 in PHEM buffer (Pipes 60 mM, Hepes 25 mM pH 6,9, EGTA 10 mM, MgCl2 2 mM), then fixed with 2% paraformaldehyde in PHEM buffer. Cells were washed in PBS (0,15 M NaCl, 10 mM Na2HPO4, 2,5 mM NaH2PO4, pH 7,2) and incubated for 15 min in PBS containing 3% BSA to prevent non-specific binding of antibodies. Primary antibody, CTS32 [4]was applied for 1 h diluted 1/20 in PBS containing 1% BSA (PBSB). Slides were washed in PBS and treated for one hour with goat anti-mouse IgG FITC conjugated antibody (GAM-FITC, Sigma) diluted 1/100 in PBSB. After three successive washes in PBS, cells were mounted in Vectashield (Vector laboratories). RNAi-induced phenotypes were observed using a DMCR epifluorescence Leica microscope equipped with a Cohu high performance CCD camera.

## Abbreviations

HCA: hydrophobic cluster analysis; GCA: generalized cluster analysis; TGD: *Tetrahymena *Genome Database; WGD: whole genome duplication; MRE: metal-response-like elements; ARE: anti-oxydant response-like elements; USE: upstream sequence elements; EST: expressed sequence tag

## Authors' contributions

RD and GB collected the data, GB, GC and PB conceived the study, designed the experiments, analyzed the data, and wrote the manuscript. SP made part of the RNAi experiments. SP, VR and BV initiated a part of this work. All authors read and approved the final manuscript.

## Supplementary Material

Additional file 1 **HCA representation of the 51 epiplasmins of *P. tetraurelia***. According to their modular organization, these proteins can be regrouped in five main groups 1 to 5. On each side of their common central domain, various modules can be identified: PVQ rich, Hinge, Y rich domains, and FLLF motif. Click here for file

Additional file 2 **Compilation of common 23 mers between epiplasmin genes**. According to RNAi knowledge, amplification of RNAi activity is mediated by the presence of small 21 to 23 mers obtained by RdRp activity. In order to estimate co-silencing possibility, the number of common 23 mers between each epiplasmin genes is presented in this table. Click here for file

Additional file 3 **Alignement of the 51 *Paramecium *epiplasmins and the four *Tetrahymena *epiplasmins (compatible fasta)**. This compatible fasta file describes the alignement of the 51 *Paramecium *epiplasmins and the four *Tetrahymena *epiplasmins. Click here for file
