# Supplementary figures and images for: Cross-study analysis of genomic data defines the ciliate multigenic epiplasmin family: strategies for functional analysis in Paramecium tetraurelia
Source: BMC Evol Biol. 2009 Jun 3;9:125. doi: 10.1186/1471-2148-9-125 (PMC2709106; doi:10.1186/1471-2148-9-125)

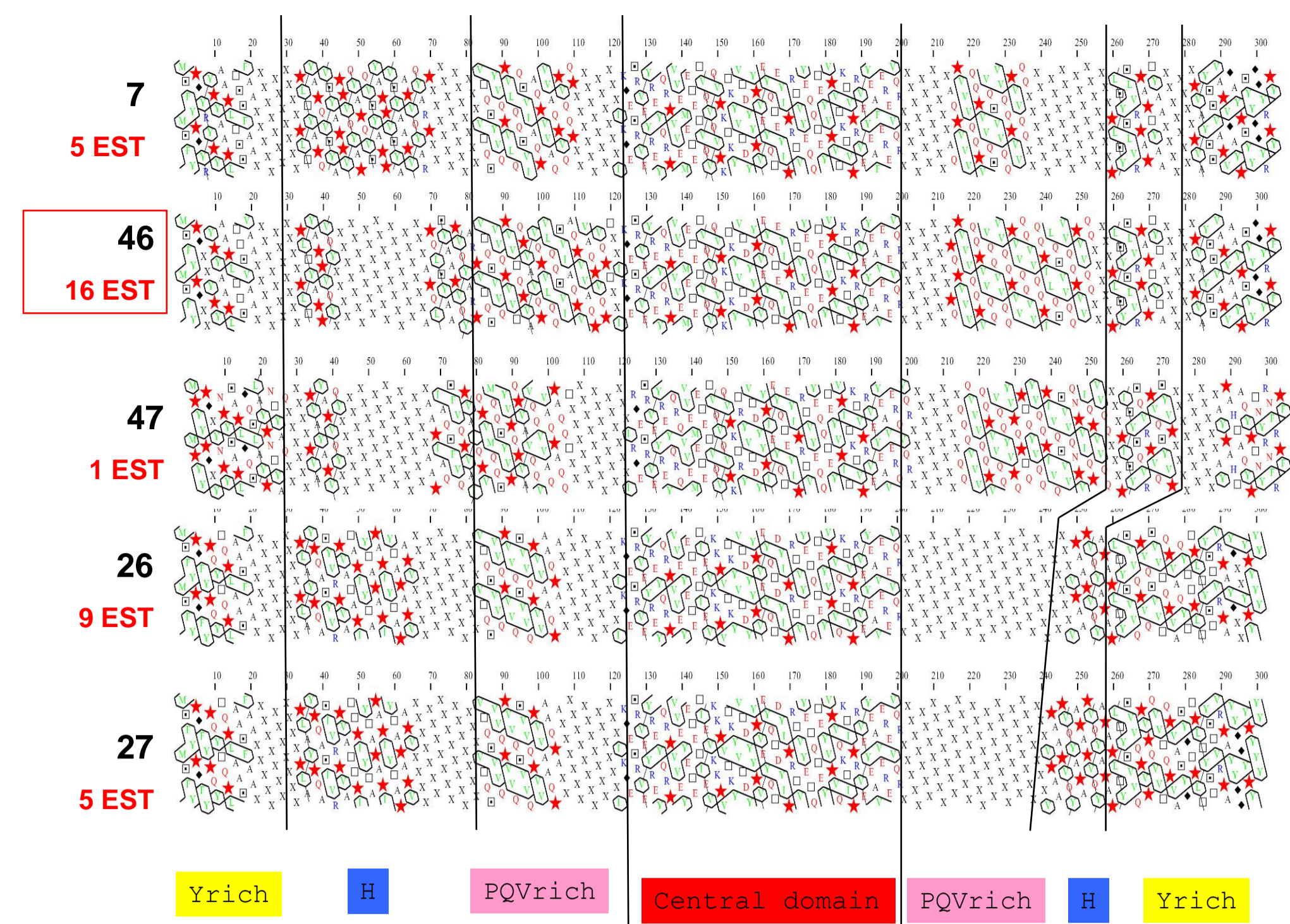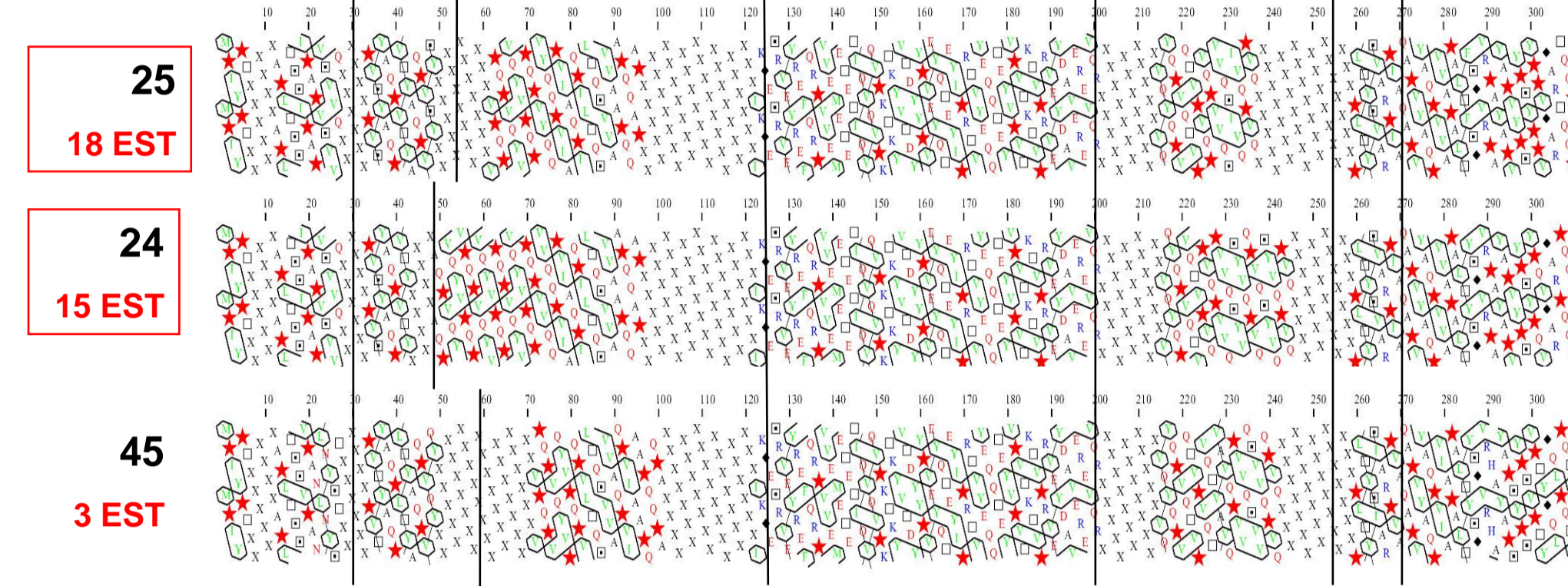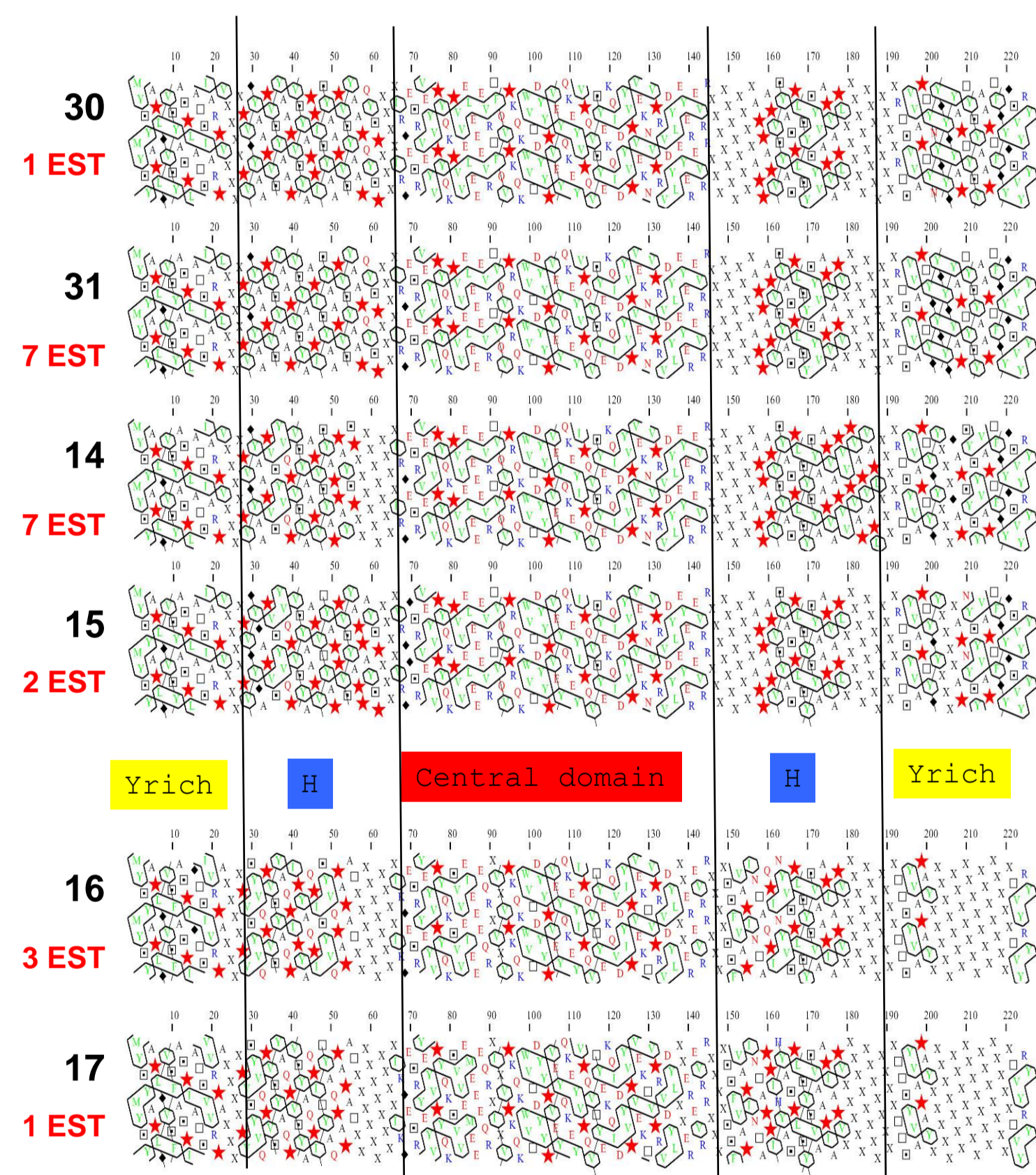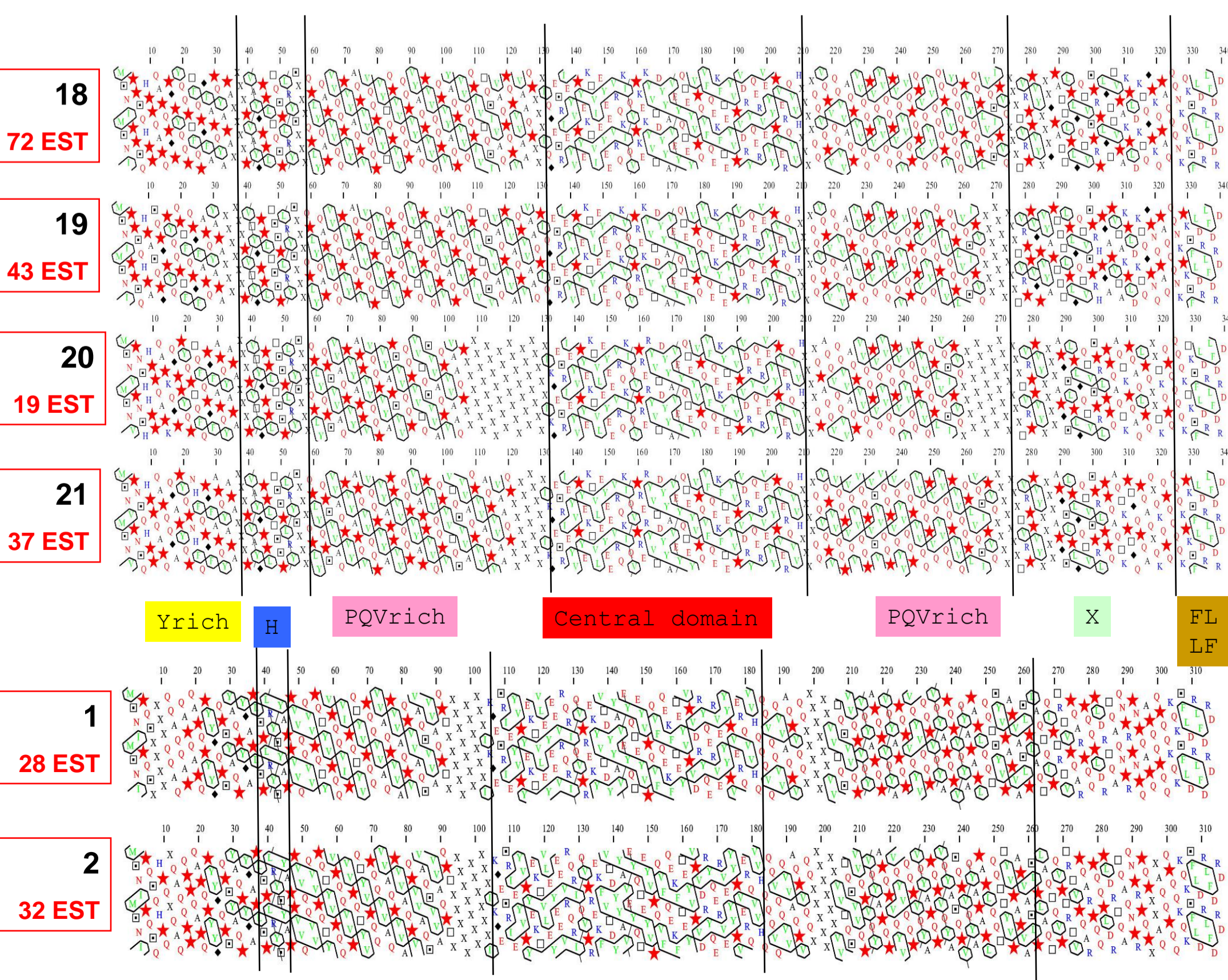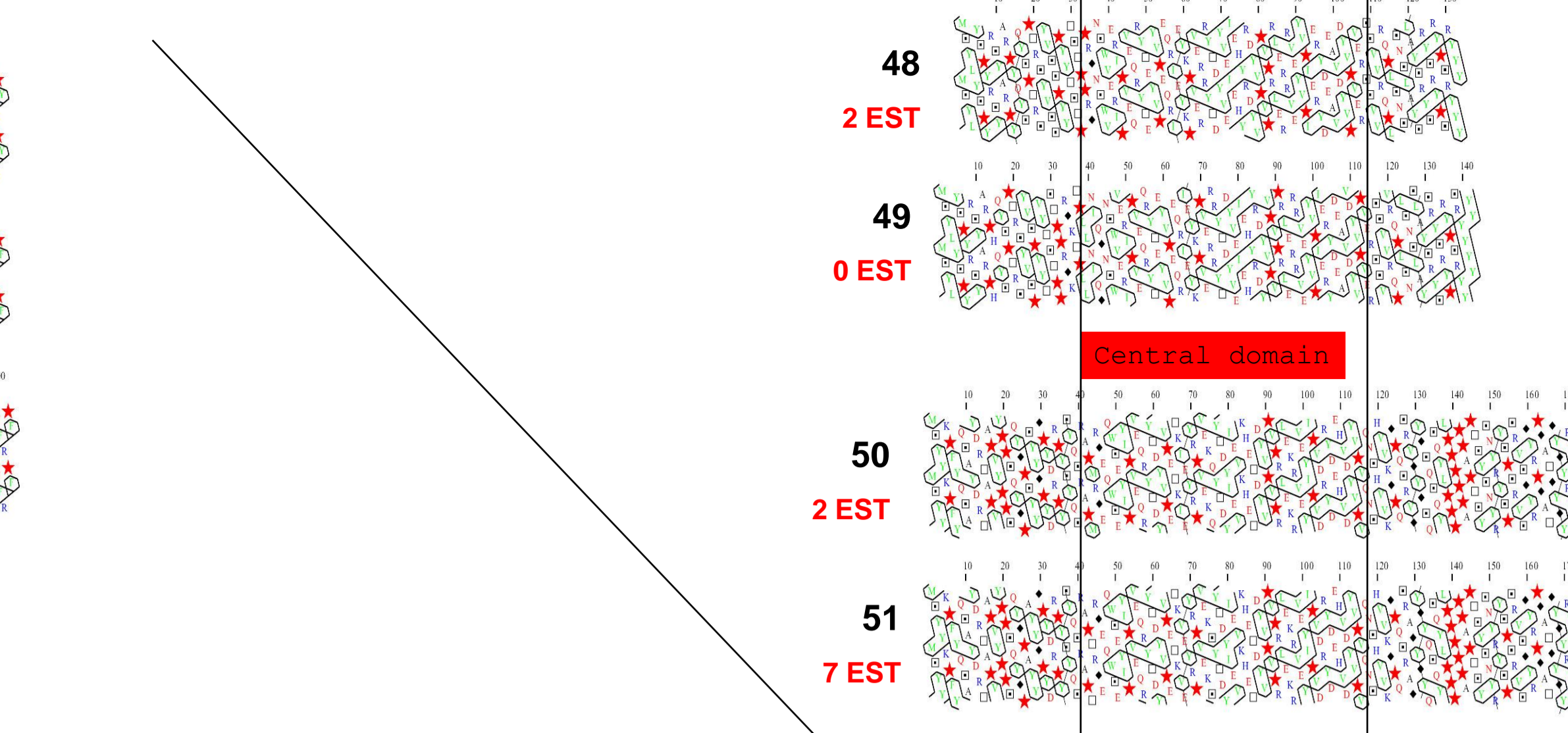

Symmetric

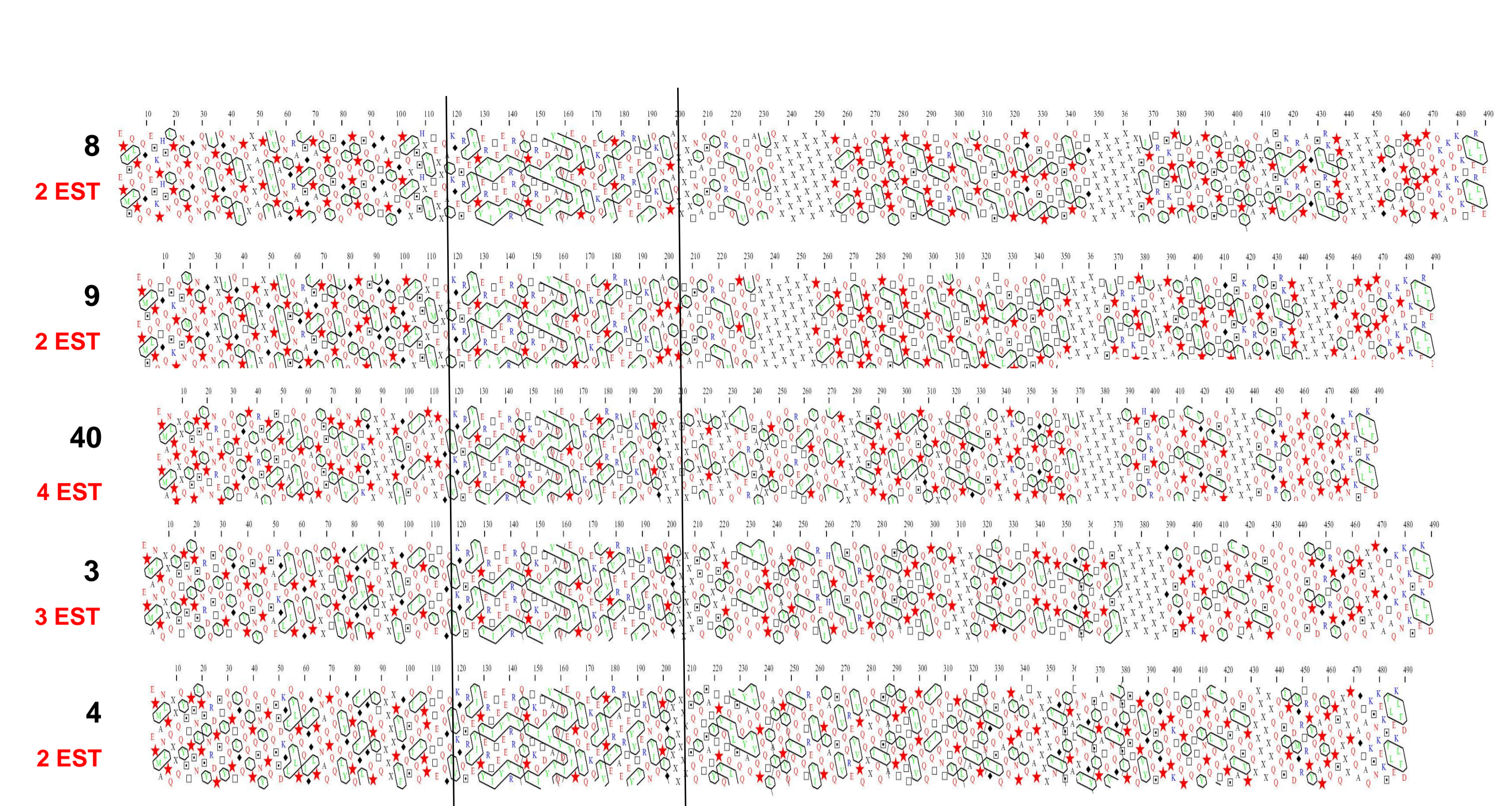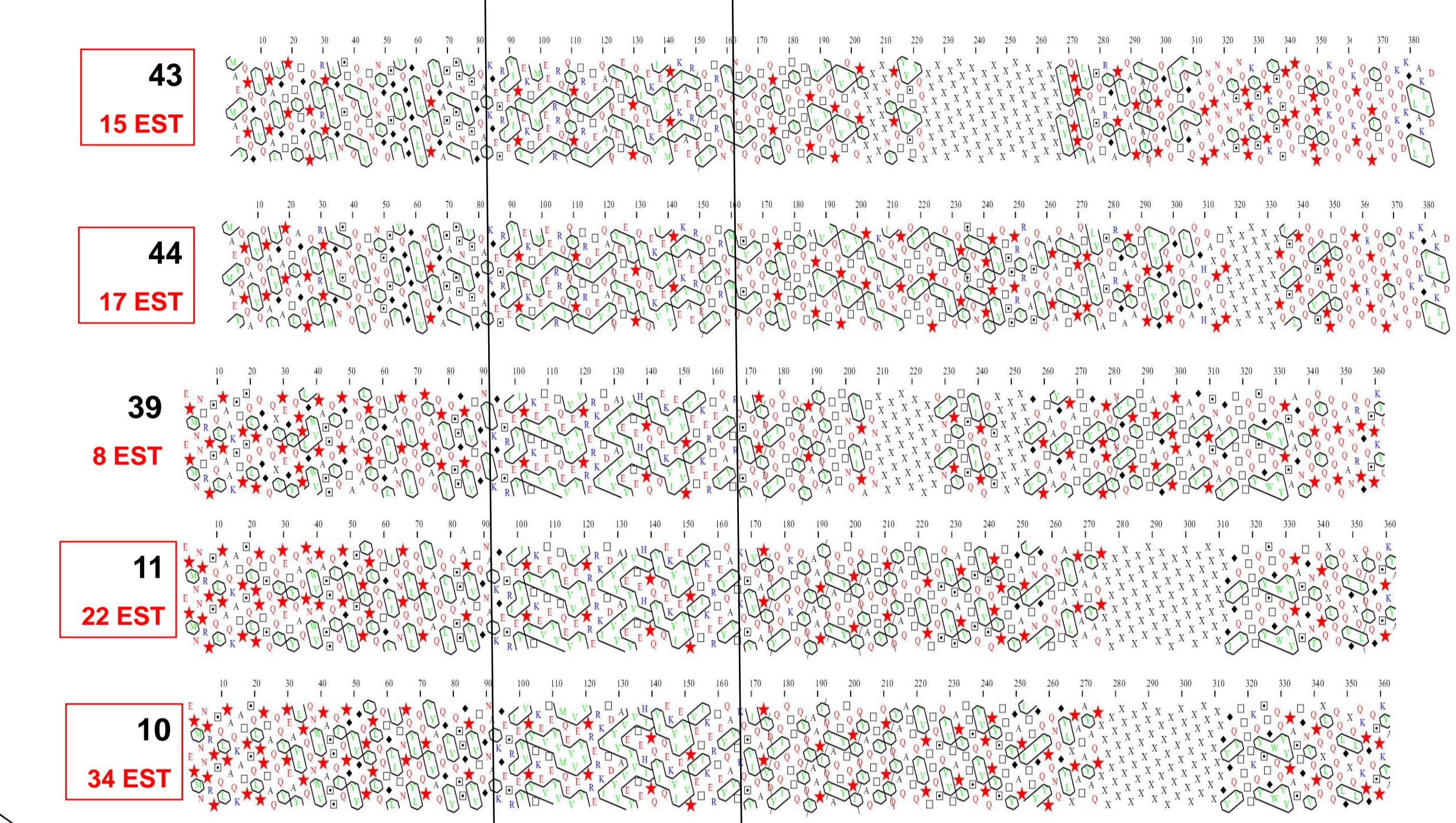

Symmetric

Atypic

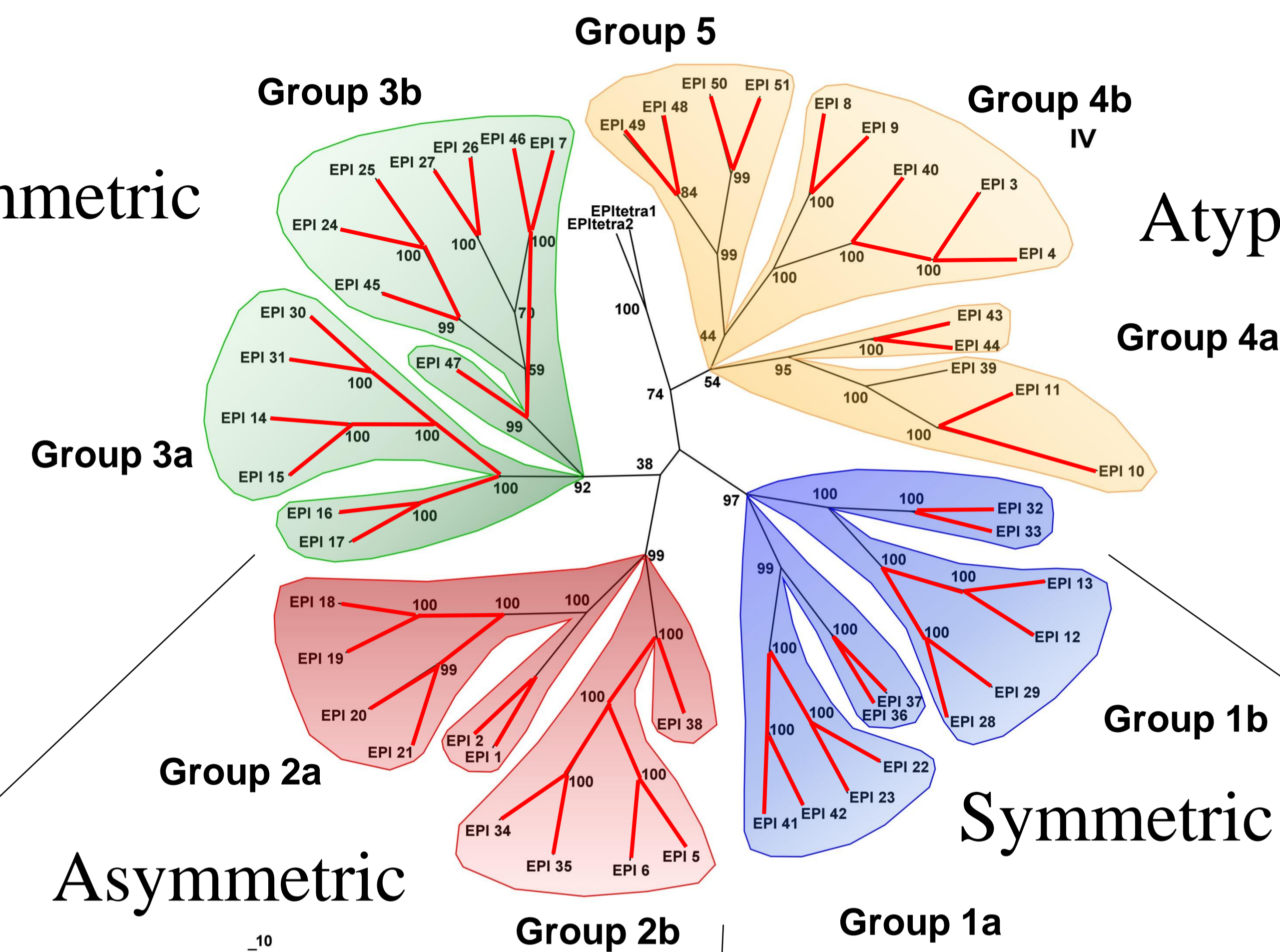

Asymmetric

Symmetric

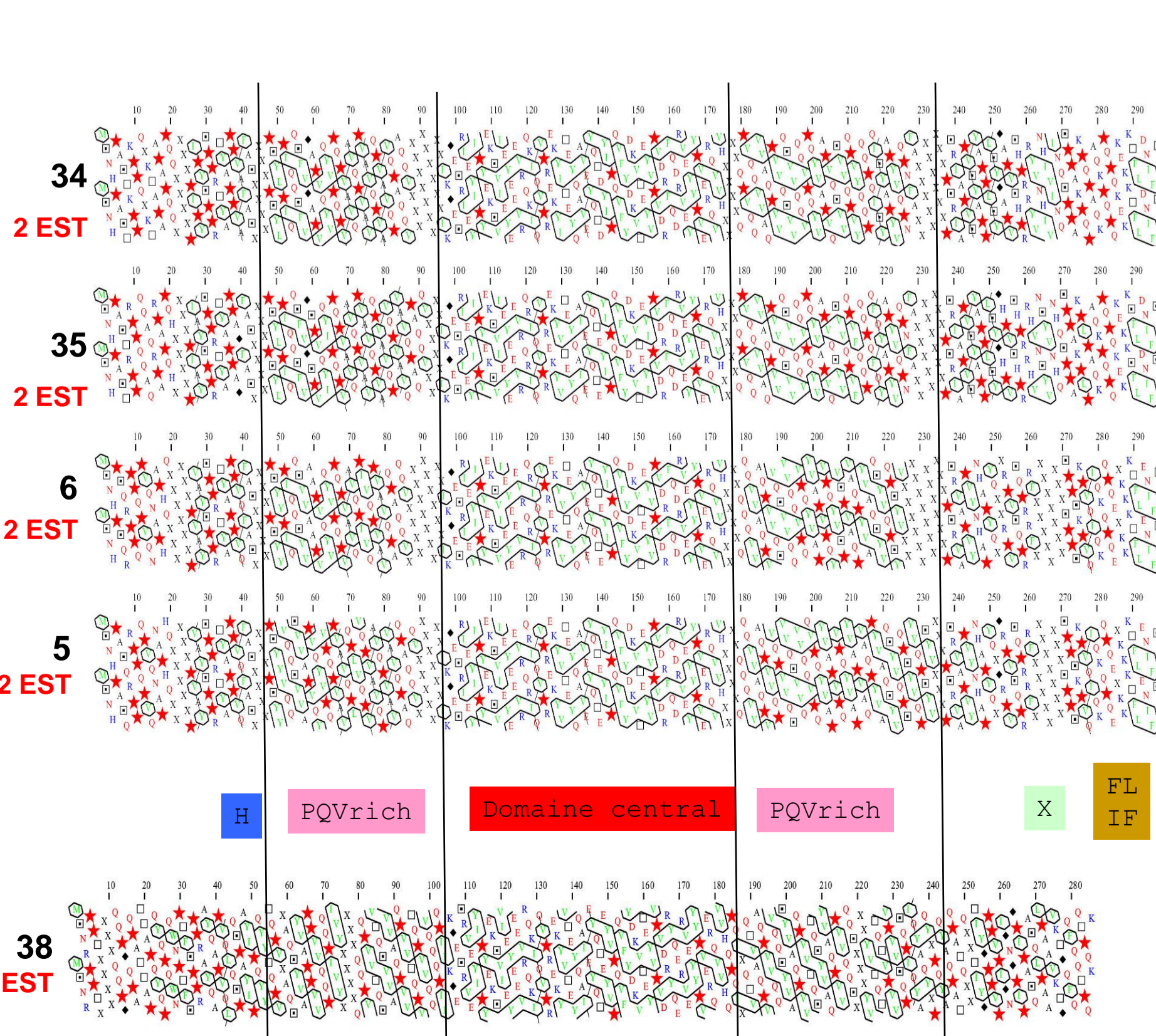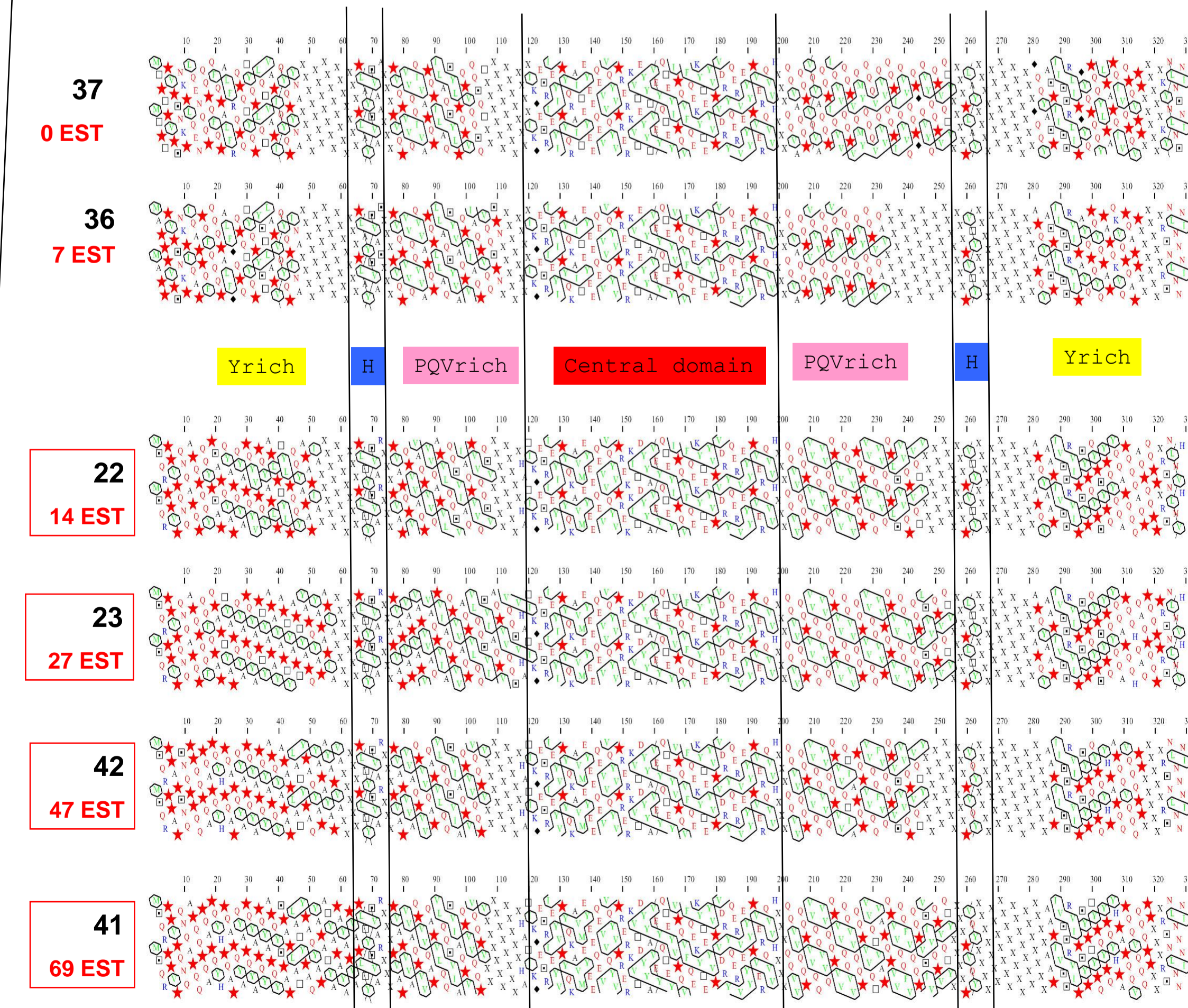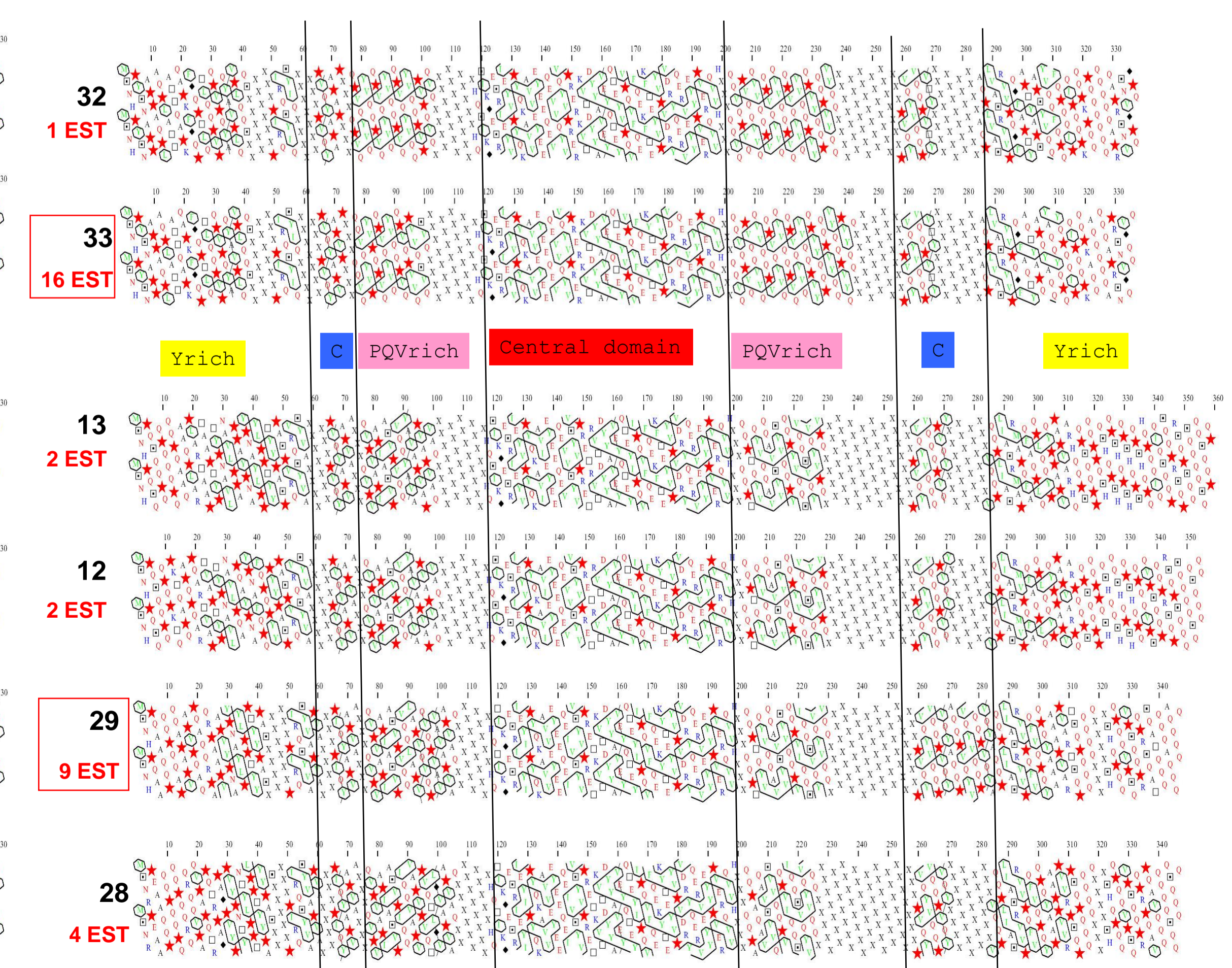

Supplement: Additional file 1 — HCA representation of the 51 epiplasmins of P. tetraurelia. According to their modular organization, these proteins can be regrouped in five main groups 1 to 5. On each side of their common central domain, various modules can be identified: PVQ rich, Hinge, Y rich domains, and FLLF motif. [file 1471-2148-9-125-S1.pdf]

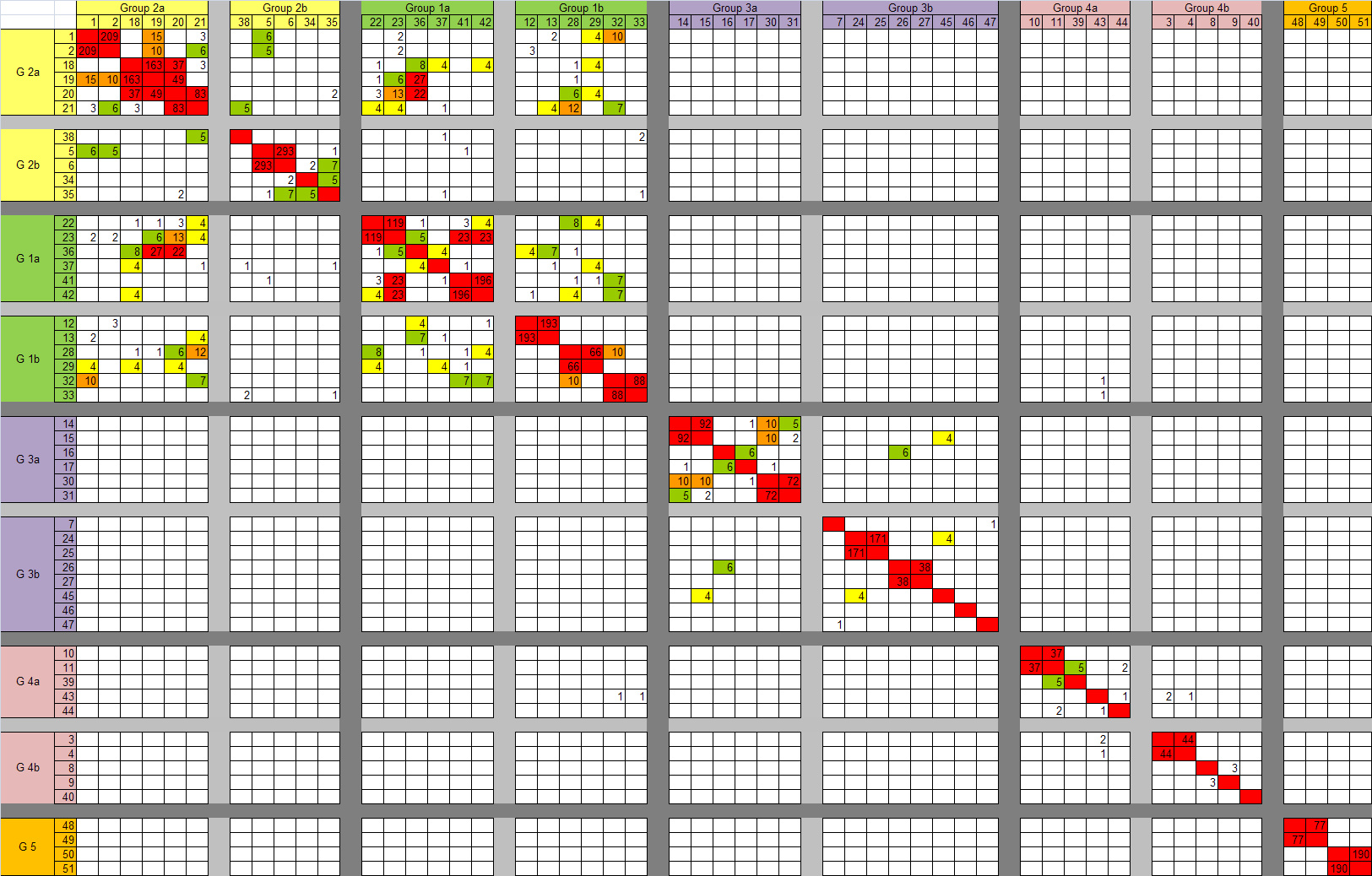

Supplement: Additional file 2 — Compilation of common 23 mers between epiplasmin genes. According to RNAi knowledge, amplification of RNAi activity is mediated by the presence of small 21 to 23 mers obtained by RdRp activity. In order to estimate co-silencing possibility, the number of common 23 mers between each epiplasmin genes is presented in this table. [file 1471-2148-9-125-S2.jpeg]
